# Digital technologies in ethnobiology and ethnoecology: methodological opportunities, ethical challenges, and participatory implications

**DOI:** 10.1186/s13002-026-00889-2

**Published:** 2026-04-26

**Authors:** Hudson Toscano Lopes Barroso da Silva

**Affiliations:** Universidade Federal Rural Do Semi-Árido (UFERSA), Mossoró, RN Brazil

**Keywords:** Ethnobiology, Ethnoecology, Digital technologies, Participatory research, Biocultural knowledge, Data sovereignty, Research ethics

## Abstract

The increasing adoption of digital technologies has reshaped methodological practices in ethnobiology and ethnoecology. Mobile data collection platforms, participatory mapping tools, biodiversity databases, remote sensing technologies, and digital analytical environments have expanded researchers’ capacity to document, integrate, and analyze biocultural knowledge across spatial and temporal scales. At the same time, digitalization raises critical methodological and ethical questions related to participation, data governance, and the representation of local knowledge systems. This article presents a critical narrative review of digital technologies used in ethnobiological and ethnoecological research, moving beyond an instrumental focus on tools to examine their methodological, ethical, and participatory implications. Digital technologies are organized into six functional categories—structured data collection, participatory mapping, biodiversity cataloging, audio recording and transcription, remote sensing, and data analysis and visualization—used as analytical devices to synthesize recurrent patterns across the literature. The review examines how these technologies shape knowledge production, integration, and communication, with particular attention to their mediating role in relationships between researchers and Indigenous peoples and local communities. Across categories, the analysis highlights trade-offs related to participation, consent, data sovereignty, standardization, and contextual depth. By synthesizing opportunities and limitations documented in empirical and methodological studies, this review argues that the value of digital technologies in ethnobiology lies less in technological novelty than in their capacity to support reflexive, transparent, and ethically grounded research practices when embedded within community-centered governance frameworks.

**Supplementary Information:**

The online version contains supplementary material available at 10.1186/s13002-026-00889-2.

## Science highlights

- Digital technologies are reshaping methodological practices in ethnobiology and ethnoecology
- Digital tools mediate how biocultural knowledge is produced, structured, and circulated
- Participatory digital methods can strengthen knowledge co-production when governance is shared
- Ethical and data governance considerations are central to responsible digital ethnobiological research
- Integrative digital approaches enhance comparability while maintaining contextual and cultural specificity

**Supplementary Information:**

The online version contains supplementary material available at 10.1186/s13002-026-00889-2.

## Introduction

Over the past two decades, ethnobiology and ethnoecology have undergone significant methodological transformations, reflecting broader shifts in how knowledge is produced, stored, and circulated in the digital age [1–4]. As fields grounded in research involving human subjects, they occupy a distinctive position at the interface between the social and environmental sciences [5, 6]. Traditionally reliant on field notebooks, analog recordings, and direct interpersonal exchange, these disciplines have long employed participatory and qualitative approaches to document local ecological knowledge [7–9].

The proliferation of digital technologies has expanded these methodological possibilities [1, 10, 11]. Mobile devices, geospatial platforms, open-access databases, and cloud-based repositories now enable the documentation of biocultural knowledge with greater precision and the integration of textual, spatial, audiovisual, and quantitative data [2, 12]. Such tools have facilitated participatory mapping, digital biodiversity documentation, and the development of interactive archives co-managed with Indigenous peoples and local communities [13–16], situating ethnobiology and ethnoecology in dialogue with broader debates on digital humanities, open science, and socio-environmental data ethics [11, 17].

Despite these advances, the adoption of digital tools remains uneven and fragmented [3, 18, 19]. In many studies, technologies are used in an instrumental manner, with limited attention to their integration into coherent methodological frameworks [4, 20, 21]. Relatively few analyses have explicitly examined digital platforms in relation to accessibility, cultural appropriateness, interoperability, or ethical safeguards within ethnobiology and ethnoecology [22, 23]. Moreover, while digitalization can enhance efficiency and reproducibility, it also introduces new asymmetries in data ownership and raises concerns about the extraction or misrepresentation of traditional knowledge—issues that resonate with broader debates in qualitative and participatory research involving human subjects [24, 25].

These challenges reveal a critical methodological gap: the limited presence of integrative perspectives that explicitly examine the mediating role of digital tools in ethnobiological knowledge production. While analogous questions regarding mediation, materiality, and the role of tools in knowledge production have been widely discussed in broader sociotechnical and organizational literatures [19, 26–28], these works do not address digital technologies in ethnobiological research directly. In this review, they are therefore mobilized as interpretative frameworks rather than as sources of field-specific conceptualization. The digital turn brings both opportunities and responsibilities, enabling the preservation of endangered languages, vernacular taxonomies, and oral histories, while simultaneously demanding reflection on who controls digital representations and who benefits from their dissemination [23, 29, 30].

Emerging technologies such as machine learning, natural language processing, and remote sensing further expand the analytical frontiers of ethnobiology and ethnoecology, often through interdisciplinary borrowings from ecology, data science, and computational social science [31–35]. Automated transcription, spatial modeling, and large-scale visualization of species–use networks are increasingly feasible [36], yet their methodological and ethical implications remain insufficiently explored [19, 37, 38]. Integrating these approaches requires interdisciplinary collaboration that balances computational innovation with ethnographic sensitivity and cultural context [39].

Digitalization also has profound implications for ethical practice and data sovereignty, as open data policies gain prominence and ethnobiological research must reconcile transparency with the protection of culturally sensitive information [19, 40, 41]. Indigenous and local data sovereignty movements emphasize that digital infrastructures are not culturally neutral, underscoring the need for protocols that ensure community control over data access, interpretation, and circulation [23, 24, 42]. Classic discussions on classification and infrastructure in science and technology studies offer important interpretative background for these debates [43].

In this context, this article critically reviews digital tools currently used in ethnobiology and ethnoecology, moving beyond an instrumental perspective to examine their methodological, ethical, and participatory implications. By comparatively analyzing how different digital technologies mediate processes of knowledge production, participation, and data governance, the review proposes an integrated analytical perspective on the role of digital infrastructures in ethnobiological and ethnoecological research practices. In doing so, the article contributes to current debates by systematically identifying recurring methodological patterns and conceptual tensions that emerge across the literature.

The review is organized into six functional categories: i) structured data collection, ii) participatory mapping, iii) biodiversity cataloging, iv) audio recording and transcription, v) remote sensing, and vi) data analysis and visualization. For each category, core functions, accessibility, and applications are discussed, highlighting how digital technologies can enhance methodological rigor, inclusivity, and reproducibility while remaining aligned with the relational and community-centered values that define ethnobiology and ethnoecology.

## Review approach and analytical strategy

This study adopts a critical narrative review approach to synthesize methodological, ethical, and participatory debates surrounding the use of digital technologies in ethnobiology and ethnoecology. Rather than pursuing exhaustive coverage or quantitative synthesis, the review prioritizes analytical depth, conceptual integration, and cross-study comparison.

Literature was identified through structured searches conducted in Web of Science and Scopus, complemented by iterative backward and forward citation tracking. The review focused on studies published between 2005 and 2025, a period corresponding to the growing integration of digital platforms, mobile devices, and geospatial tools into ethnobiological research [1, 2]. Searches combined terms related to ethnobiology and ethnoecology with terms referring to digital technologies, as detailed in Table 1. Search terms were applied to titles, abstracts, and keywords when available.

**Table 1:** Search strategy used to identify studies in Web of Science and Scopus

Field of study	("ethnobiology" OR "ethnoecology" OR "ethnobotany" OR "traditional ecological knowledge" OR "local ecological knowledge" OR "biocultural knowledge")
Digital technologies	("digital tool*" OR "digital technolog*" OR "mobile data collection" OR "geographic information system*" OR "GIS" OR "participatory mapping" OR "remote sensing" OR "digital database*" OR "data visualization" OR "citizen science platform*" OR "mobile application*" OR "open data" OR "biodiversity informatics" OR "community-based monitoring")

The combined search returned 476 records across both databases, of which 172 were identified as duplicates and removed, yielding 304 unique records for screening. Title and abstract screening against the inclusion criteria resulted in 215 records retained for full-text assessment.

Studies were included if they met the following criteria: i) were peer-reviewed publications; ii) explicitly reported, analyzed, or critically discussed the use of digital technologies within ethnobiological or ethnoecological research contexts; and iii) engaged with methodological, ethical, or participatory dimensions of digital research practices. Publications were excluded when digital tools were mentioned only incidentally, when the focus was purely technical without substantive engagement with ethnobiological or ethnoecological questions, or when the material consisted of editorials, opinion pieces, or other non-peer-reviewed sources. These criteria were applied during title and abstract screening, followed by full-text assessment when necessary.

Of the 215 records retained for full-text assessment, 51 studies met the inclusion criteria as primary empirical sources identified through the structured search. An additional 28 studies were incorporated through iterative backward and forward citation tracking of the included references, a strategy that proved essential for recovering literature published in participatory GIS, community-based monitoring, Indigenous data governance, and biodiversity informatics — fields whose indexing overlaps only partially with the ethnobiology-specific search terms employed. The final corpus comprises 79 empirical and methodological studies from ethnobiology, ethnoecology, and closely related fields.

The classification of digital technologies into functional categories was based on their primary role within the research process rather than on technological form or disciplinary boundaries. Categories were defined according to the dominant function performed by each technology (e.g., data generation, spatial representation, analytical processing, or knowledge dissemination), while recognizing that individual tools may operate across multiple stages of research. Potential overlaps among categories were therefore treated as analytically informative rather than as classification limitations, allowing the review to examine how different digital functions intersect and reconfigure participation, ethical risk, and epistemological mediation.

To support comparative synthesis across heterogeneous studies, the categorized literature was subsequently re-examined to identify recurring analytical patterns related to participation, ethical risk, data governance, and epistemological mediation. These dimensions were not treated as fixed coding categories but emerged through successive readings and cross-study comparison [25, 44]. This interpretative approach allowed the review to move beyond descriptive classification and to examine how different types of digital technologies shape the social and epistemological conditions under which ethnobiological knowledge is produced, interpreted, and circulated.

## Digital technologies in ethnobiology and ethnoecology

Digital technologies have become increasingly embedded in contemporary ethnobiological and ethnoecological research, supporting the systematic collection, organization, and analysis of socio-ecological data across diverse research contexts [3, 10, 11, 19, 45, 46]. Their adoption is consistently associated with expanded capacities to integrate cultural, ecological, and spatial information within participatory research designs, particularly in studies that combine ethnographic, spatial, and ecological data streams [12, 16]. Beyond data management, digital tools actively shape how knowledge is produced, interpreted, and circulated through interactions among researchers, Indigenous peoples and local communities, with outcomes varying according to local sociopolitical contexts, institutional arrangements, and governance frameworks [27, 28].

In this review, rather than treating digital technologies as neutral instruments or invoking mediation as a metaphor for technological influence, mediation is employed as an analytical lens to examine how digital infrastructures reconfigure relations of agency, visibility, and control within ethnobiological and ethnoecological knowledge production [26, 28].

This section introduces analytically defined functional categories of digital technologies used in ethnobiology and ethnoecology that structure the review. Their purpose is not to establish rigid typologies, but rather to provide a comparative scaffold for examining how different technological functions are associated with distinct configurations of participation, ethical risk, and epistemological mediation.

**Fig. 1:**
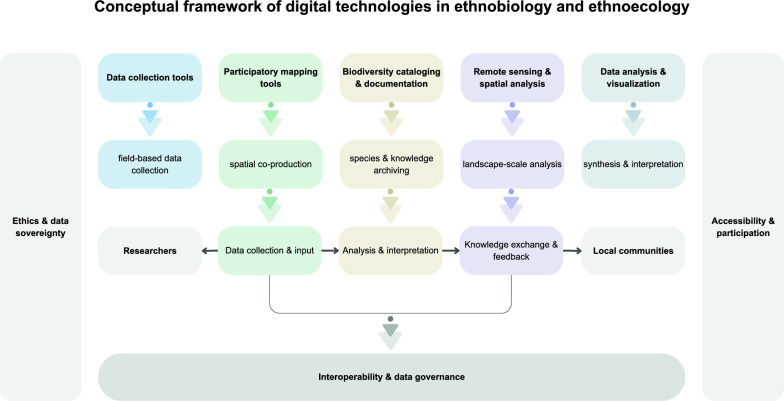
Conceptual framework illustrating the main functional categories of digital technologies used in ethnobiology and ethnoecology and their roles across the research process, highlighting cross-cutting dimensions related to ethics and data sovereignty, accessibility and participation, and interoperability and data governance

As illustrated in Fig. 1, this framework organizes the discussion by linking functional categories of digital technologies to cross-cutting methodological and ethical dimensions [47], enabling systematic comparison across studies. Table 2 summarizes the main functional categories identified in the literature and synthesizes their associated methodological opportunities and ethical challenges.

**Table 2:** Overview of the main functional categories of digital technologies used in ethnobiology and ethnoecology, organized according to research stage, primary data types, levels of participation, methodological opportunities, and associated ethical and practical challenges

Functional category	Main research stage	Examples of tools	Primary data types	Level of participation	Key methodological opportunities	Main ethical and practical challenges
Data collection tools	Field-based data collection	KoBoToolbox, ODK Collect, Epicollect5	Quantitative, qualitative, spatial (text, images, audio, GPS)	Medium–High	Standardized data capture; real-time integration of socio-ecological data; improved comparability across sites	Informed consent; data privacy; long-term storage of sensitive cultural information
Participatory mapping	Spatial data generation and validation	QField, Maptionnaire, GPS Essentials	Spatial, qualitative, participatory inputs	High	Co-production of spatial knowledge; visualization of local territorialities; dialogical research processes	Sensitivity of spatial data; risks of misuse; ownership and control of territorial information
Biodiversity cataloging and documentation	Species recording and knowledge archiving	iNaturalist, Audio Memos, Transkriptor	Images, audio, species records, narratives	Medium	Integration of scientific and vernacular taxonomies; creation of digital biocultural archives	Intellectual property; exposure of culturally restricted or ecologically sensitive information
Remote sensing and spatial analysis	Landscape-scale analysis and monitoring	Drones, Sentinel, Landsat	Raster imagery, environmental indicators	Low–Medium	Linking local knowledge with landscape-level patterns; temporal monitoring of environmental change	Scale mismatches; abstraction of cultural meanings; privacy and surveillance concerns
Data analysis and visualization	Analysis, synthesis, and dissemination	R, Python, GIS platforms, dashboards	Multivariate, textual, spatial, network data	Medium	Mixed-methods integration; transparency and reproducibility; participatory interpretation of results	Representation bias; oversimplification of local realities; data governance and access control

An overview of the main digital tools discussed in this review, organized by functional category and primary applications, is provided in Supplementary Material S1.

### Data collection tools

Mobile and web-based data collection platforms are widely used in contemporary ethnobiological fieldwork, particularly in studies that seek to systematize the collection of quantitative, qualitative, and spatial information in situ [14, 48, 49]. These platforms are primarily used to structure field interactions through standardized digital forms capable of integrating multiple types of data in real time [50, 51].

Widely used systems such as KoBoToolbox, Open Data Kit (ODK) Collect, and Epicollect5 provide open-source and customizable environments for data entry under offline or low-connectivity conditions. In ethnobiological and ethnoecological research, these platforms enable the integration of ethnographic narratives and ecological observations within unified data structures, allowing researchers to associate vernacular and scientific species names, record management practices, and georeference sites of ecological interaction [52, 53].

Linking socio-cultural narratives to spatially explicit data is frequently reported as enhancing analytical depth, particularly in studies exploring biocultural patterns, resource distribution, and environmental change [54, 55]. At the same time, these integrations are shaped by methodological decisions related to questionnaire design, data categorization, interface constraints, and control over data entry [48]. Such decisions influence whose knowledge is rendered visible, how it is translated into standardized variables, and which forms of knowledge remain peripheral or excluded from digital representation [13, 43, 56].

Beyond gains in efficiency, digital data collection platforms are often embedded within participatory research designs, particularly when community members are involved in data entry, validation, or data management [21, 57]. However, studies document internal differences within communities, including unequal access to devices, gendered and generational divisions in data entry roles, and contestation over who defines data categories and controls digital storage infrastructures [13, 58–60].

Empirical examples from community-based monitoring and ethnobotanical research illustrate how digital platforms have been used to record species occurrences, land-use practices, and environmental change indicators, improving data consistency while requiring negotiated decisions about categories and validation procedures [61–63]. At the same time, the integration of local knowledge into standardized digital databases has enabled large-scale comparative analyses while highlighting tensions between analytical standardization and the contextual richness of vernacular classifications [64–66]. Similar patterns are reported in Indigenous environmental monitoring initiatives, where data entry protocols, interface design, and governance arrangements shape participation and authority within monitoring processes [41, 59, 63], often revealing internal differences in who participates in data collection, how knowledge is validated, and which actors exercise authority over the interpretation and circulation of digital records.

The adoption of digital data collection tools also raises persistent ethical and contextual concerns related to informed consent, data privacy, and the long-term storage of sensitive cultural information are repeatedly identified as central methodological challenges rather than ancillary considerations [41, 67, 68]. Without explicit governance arrangements, digital infrastructures may amplify existing power asymmetries or expose culturally restricted knowledge systems [13, 17]. When embedded within reflexive and participatory research frameworks, digital data collection technologies can support analytical rigor and cross-scale integration [69], but maintaining contextual grounding requires sustained attention to how digital forms structure interaction, representation, and authority throughout the research process [70, 71].

Accessibility constraints remain a recurring limitation, and while offline-capable platforms can mitigate connectivity barriers [49, 72], they do not eliminate challenges related to device availability, maintenance, data synchronization, and dependence on external technical support [73, 74]. Mitigation strategies include shared device use, simplified form design, local data storage, and iterative training processes [63, 75].

### Participatory mapping

Participatory mapping is extensively documented in ethnobiology and ethnoecology as a methodological approach that introduces a spatial dimension to local ecological knowledge and supports the articulation of human–environment relationships [9, 15, 16]. It is primarily employed in studies addressing territorial use, resource distribution, and culturally meaningful landscapes, where digital mapping tools are combined with collaborative fieldwork practices [76, 77]. Beyond cartographic outputs, participatory mapping is examined here as a political and epistemological practice, as it shapes which forms of territorial knowledge become visible, to whom, and under what institutional and governance conditions [78, 79].

Several digital platforms are increasingly used in participatory mapping initiatives, including QField, Maptionnaire, and GPS-based mobile applications, particularly in studies conducted under low-connectivity conditions where field-based data collection must later be synchronized with existing GIS projects once connectivity becomes available [49, 51, 80]. These tools are also widely employed in participatory spatial surveys and community-based mapping initiatives to document perceptions, values, and lived experiences, as well as to record locations, tracks, and photographs during field activities [16, 81].

Across ethnobiological case studies, these tools are used to document areas of traditional use, sacred sites, and zones under ecological pressure, often in connection with conservation planning, territorial claims, or community-led management initiatives [68, 82]. In this context, participatory maps are frequently interpreted as boundary objects that mediate between spatial ecological data and social narratives related to territory, identity, and environmental change [14, 64]. This interpretation draws on broader discussions of boundary objects as analytical devices used to understand coordination across social worlds, rather than as direct theoretical accounts of participatory mapping itself [83].

Case-based analyses documenting the mapping of customary territories, sacred sites, or resource-use areas show that digital maps can support political recognition, community planning, and intergenerational knowledge transmission [15, 84, 85]. However, studies consistently document internal contestations within communities, including disputes over who participates in mapping activities, who controls mapping devices, how territorial categories are defined, and whose knowledge is included or excluded from final representations [78, 86]. These findings indicate that participatory mapping processes are shaped by local power relations, institutional mediation, and negotiations over data access, ownership, and circulation, illustrating how digital mapping technologies actively mediate the production and representation of territorial knowledge [48, 85, 87].

Methodologically, participatory mapping supports reflexive and dialogical research designs in which maps are treated as iterative processes rather than static products [88], with digital platforms enabling iterative cycles of validation, collective interpretation, and revision [13]. However, the literature stresses that this approach raises acute ethical and practical challenges, particularly when spatial information about culturally significant sites or customary territories becomes vulnerable to misuse as data circulate beyond agreed governance frameworks, underscoring the need for explicit agreements on storage, access, and long-term control [40, 84].

Accessibility constraints further shape participatory mapping outcomes, as empirical studies note barriers related to uneven access to devices, differential familiarity with digital interfaces, language limitations, and the institutional costs associated with data processing and long-term storage [86, 89], leading to mitigation strategies such as shared device use, facilitated mapping sessions, simplified interfaces, and collective interpretation workshops [84].

When embedded within ethically grounded and context-sensitive research frameworks, participatory mapping contributes to biocultural conservation efforts by supporting collective reflection on landscape change and strengthening local engagement in environmental decision-making [15, 90]. At the same time, the studies emphasize that the analytical and political value of participatory mapping depends on sustained attention to internal community dynamics, institutional arrangements, and governance mechanisms, rather than on the mere adoption of digital mapping technologies [17, 59].

### Biodiversity cataloging and documentation

Digital platforms for biodiversity cataloging and documentation differ from field-based data collection tools in that they primarily function as infrastructures for organizing, curating, and circulating biodiversity records and associated cultural information across broader analytical and institutional contexts [46]. Across the literature, these tools are primarily applied in studies seeking to document species use, local classifications, and ecological observations in formats that can be integrated with scientific databases [10, 45]. By enabling the co-documentation of species records and cultural information, such platforms are frequently described as facilitating dialogue between scientific taxonomies and vernacular classification systems, while also introducing new forms of standardization and visibility [91, 92].

Beyond data management, biodiversity cataloging platforms are examined here as digital infrastructures that mediate how biocultural knowledge is archived, rendered interoperable, and circulated across institutional and spatial scales [3, 19]. In this sense, they function as digital archives of biocultural heritage, shaping not only what kinds of knowledge are preserved, but also how knowledge is formatted, classified, and made accessible beyond local contexts [30, 93]. This mediating role is not treated as an inherent property of technology, but as an outcome of governance arrangements, metadata standards, and access protocols embedded in specific platforms [11, 41].

Among the platforms most frequently discussed in the studies are iNaturalist, Audio Memos, and Transkriptor. iNaturalist enables users to upload georeferenced images for species identification and has been widely adopted in initiatives that integrate culturally significant species into broader biodiversity datasets [45, 94]. Empirical studies show that, in ethnobiological contexts, the platform supports the documentation of local taxonomies, species uses, and ecological observations alongside scientific classifications, while also raising concerns related to data ownership, downstream data reuse, and the potential recontextualization of culturally embedded knowledge once records enter global open-access infrastructures [95–98].

Complementing visual documentation, Audio Memos and Transkriptor are primarily used to record and process oral data, including interviews, narratives, and soundscapes [25, 58]. Studies employing these tools emphasize their role in reducing the time required for transcription and data organization, thereby facilitating the creation of structured and searchable archives that link biological information with oral histories and ethnographic observations [42, 44]. More recent applications point to the growing use of automated transcription and speech-recognition systems, often based on machine learning, which introduce additional layers of mediation related to language recognition accuracy, algorithmic bias, and the treatment of minority or Indigenous languages [33, 35, 37]. Empirical accounts further highlight variation in who controls recording devices, who authorizes transcription, and how recorded narratives are stored or shared, underscoring negotiated practices of consent, circulation, and authorship [99, 100].

Applications of digital cataloging platforms documented across the literature include cultural inventories of species used for food, medicine, rituals, or construction, as well as the organization of qualitative narratives linked to specific taxa or ecological contexts [4, 101]. By enabling cross-referencing between species records and ethnographic data, these tools support mixed-method analyses that combine ecological breadth with ethnographic depth [102, 103]. Studies indicates, however, that such integrations are strongly shaped by methodological decisions regarding data standards, metadata design, language representation, and access permissions, which influence whose knowledge becomes visible and how it is contextualized [19, 43].

Biodiversity cataloging platforms consistently reveal tensions between scientific visibility and community control over knowledge [23, 24], and while open-access databases facilitate cross-scale integration of species records and increase the visibility of culturally significant taxa [45, 46, 65], they may also expose sensitive ecological and cultural information when governance arrangements are insufficiently defined [40, 104]. Empirical studies also highlight that decisions about what knowledge is recorded, shared, or restricted may reflect internal governance arrangements and power asymmetries within communities, including differences between knowledge holders, community leaders, and external collaborators [22, 24, 40, 41]. Studies addressing these dynamics emphasize that issues of intellectual property, benefit sharing, and data sovereignty are not ancillary concerns but central methodological challenges shaping how biodiversity documentation is designed, accessed, and mobilized [41, 90].

Accessibility constraints further condition the use of digital biodiversity platforms, as studies report barriers related to limited internet connectivity, uneven access to smartphones or cameras, language constraints, and the technical skills required to navigate digital interfaces [51, 72, 75]. Mitigation strategies include offline data collection modes, community-based data stewards, shared devices, and the use of locally hosted or restricted-access repositories [41, 57].

Digital biodiversity documentation therefore raises persistent ethical concerns related to data governance and intellectual property [23, 40]. Open-access platforms may inadvertently expose sensitive information, such as the locations of rare species or culturally restricted resources, particularly when data circulate beyond the original research context [105]. Similarly, the recording, transcription, and storage of interviews require careful attention to consent, privacy, and culturally appropriate protocols for knowledge circulation [17, 67]. Addressing these challenges, as emphasized across the studies, depends on collaborative agreements that ensure community control over data access, interpretation, and dissemination [30, 106].

In ethically grounded participatory research contexts, digital biodiversity cataloging platforms can support both scientific synthesis and cultural resilience [90, 107]. By enabling the integration of multiple knowledge systems and supporting community-led monitoring and educational initiatives, these tools contribute to a more reflexive ethnobiology [64, 65]. Their analytical value, however, depends less on technological capacity than on the governance, contextualization, and negotiation of digital archives across local, institutional, and scientific domains [69, 108].

### Remote sensing and spatial analysis

Remote sensing and spatial analysis are increasingly incorporated into ethnobiology and ethnoecology as approaches that expand the spatial and temporal scales at which human–environment interactions can be examined [31, 109, 110]. These methods are primarily employed to relate fine-grained ethnographic observations to landscape-level patterns, supporting integrative analyses that connect cultural perceptions, land use, and ecological processes [71, 111]. By providing synoptic and temporal views of vegetation dynamics and land-cover change, spatial technologies enable the examination of environmental transformations that affect biocultural systems across broader spatial extents [65, 107, 112].

A range of tools is used to integrate ethnographic data with spatially explicit environmental information, as studies document the use of drones to generate high-resolution imagery at spatial scales meaningful within specific community contexts, often complementing participatory mapping and field-based observations [113, 114]. At broader spatial scales, satellite imagery from platforms such as Landsat and Sentinel supports long-term ecosystem monitoring and is commonly analyzed using open-source environments for spatial modeling, statistical analysis, and visualization [115, 116].

Recent studies report the growing incorporation of machine learning–based techniques for image classification, land-cover mapping, and change detection in ethnobiological and ethnoecological research, particularly in analyses that link spatial environmental dynamics with local ecological knowledge and socioecological observations [32, 34, 117, 118]. These approaches are used to automate the processing of large spatial datasets [119], identify patterns in vegetation dynamics [120, 121], and generate temporal indicators that can be related to ethnographic observations and local environmental narratives [92, 122, 123]. While such applications expand analytical capacity and efficiency, the literature emphasizes that algorithmic workflows remain analytically opaque for most community participants and may further centralize interpretative authority if not accompanied by participatory validation and transparent modeling choices [11, 37].

Within ethnobiological research, remote sensing and spatial analysis are increasingly used to examine vegetation dynamics in areas of traditional resource use, map socio-ecological interactions, and relate environmental change to local perceptions and livelihoods [31, 113]. Studies integrating satellite-derived indicators with ethnographic data show how spatial patterns of land-cover change, vegetation dynamics, or hydrological variability can be related to shifts in subsistence practices, well-being, or knowledge transmission, particularly in contexts characterized by environmental variability [109, 122, 123].

While these applications expand the analytical scope of ethnobiological research, they also introduce methodological and epistemological tensions. On the one hand, satellite- and drone-derived indicators—often processed through machine learning pipelines—enable cross-scale integration, temporal comparison, and pattern detection beyond what is possible through field-based methods alone [117, 119]. On the other hand, multiple studies caution that these approaches may introduce epistemological distance when analytical control remains concentrated within research teams and when interpretative processes are weakly connected to local knowledge holders [11, 19]. Under such conditions, landscapes risk being rendered as abstract analytical surfaces rather than as culturally situated territories [65, 68]. Empirical studies also note that decisions about interpretation and validation may involve differentiated participation within communities, reflecting local governance arrangements and asymmetries in access to technical expertise and spatial knowledge [54, 63, 101].

Several studies document attempts to mitigate this distance by combining remote sensing with participatory mapping, community-based dialogue, and collective interpretation of spatial outputs [124, 125]. In applied contexts, visual materials derived from satellite imagery, drones, or classified land-cover maps are used to support collective reflection on land-use histories, environmental degradation, or conservation planning, facilitating communication among community members, researchers, and decision-makers [13, 84, 114]. However, studies indicate that the effectiveness of such integrative approaches is shaped by institutional arrangements, unequal access to analytical infrastructure, and the extent to which interpretative authority over spatial outputs is genuinely shared [47, 69, 126].

Accessibility and connectivity constraints are consistently identified as persistent limitations in the application of remote sensing within ethnobiological research, as several studies document challenges related to limited internet access [51, 72], restricted computational resources [127], and dependence on externally hosted platforms for data processing [3, 19]. Mitigation strategies discussed in the literature include the use of offline-capable software, local data storage, low-bandwidth visualization formats, and the translation of complex spatial outputs into simplified maps or printed materials for collective discussion [84, 128]. While these strategies do not eliminate structural inequalities, they are presented as pragmatic adaptations that partially reduce barriers to participation.

**Fig. 2:**
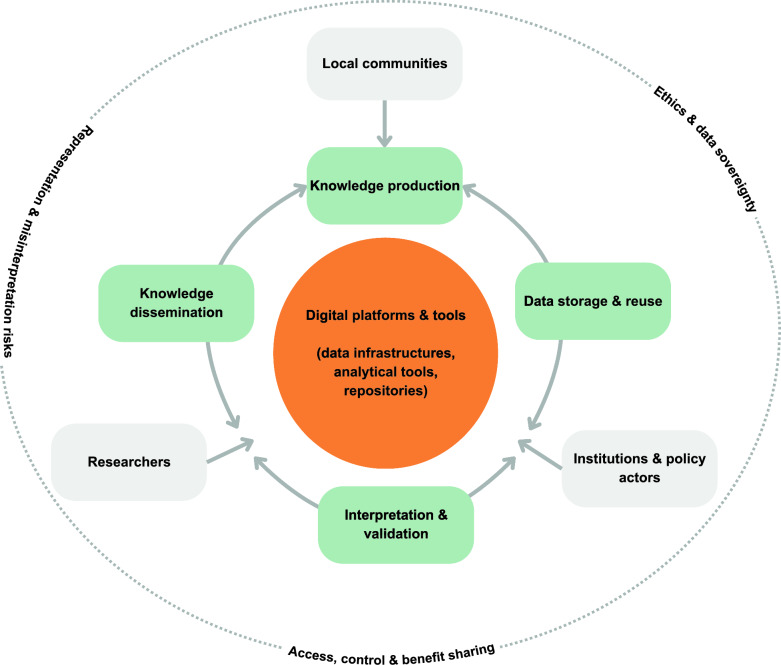
Schematic representation of the mediating role of digital technologies in the co-production, circulation, and reuse of ethnobiological knowledge. The figure highlights interactions among researchers, local communities, and digital infrastructures, as well as key ethical tensions related to data sovereignty, representation, and benefit sharing

The use of remote sensing in ethnobiology therefore requires sustained critical attention to scale, interpretation, and ethics [129–131]. Studies highlight concerns related to privacy, consent, and surveillance, particularly in relation to drone-based data collection and the remote generation of spatial information [130, 131]. More broadly, the integration of remote sensing and machine learning–based spatial analysis into ethnobiological research requires approaches that are both technically robust and socially responsive [47, 64]. Ensuring that spatial data support co-produced understandings of environmental change depends on explicit governance arrangements, participatory interpretation, and alignment with community-defined priorities, rather than on analytical capacity alone [40, 126] (Fig. 2).

### Data analysis and visualization

The increasing volume and complexity of data generated in ethnobiological and ethnoecological research have led to the growing use of digital tools for data analysis and visualization, particularly in studies combining ecological, cultural, and spatial information [10, 109]. Across the literature, these tools are primarily employed to explore multidimensional relationships among variables and to transform heterogeneous datasets into interpretable analytical outputs concerning human–environment interactions [46, 110, 111]. When critically applied, analytical and visualization tools are described as supporting transparency, reproducibility, and interdisciplinary dialogue, while simultaneously raising questions about representation, abstraction, and interpretative authority [19, 45, 127].

Open-source analytical environments such as R and Python are widely used as core analytical infrastructures for statistical modeling, text analysis, network analysis, and spatial data processing [116, 132]. Studies show that these platforms facilitate mixed-method analytical workflows by enabling the integration of quantitative ecological metrics with qualitative or categorical ethnographic data [102, 129, 133]. Such integrations are used to examine relationships between species knowledge, cultural values, environmental conditions, and governance arrangements across multiple spatial and temporal scales [90, 107].

Recent studies also point to the increasing incorporation of automated and semi-automated analytical techniques, including machine learning approaches for text processing [134], pattern recognition [33], and classification of large datasets [34, 135]. These methods are applied in tasks such as automated coding of interview transcripts, clustering of species–use networks, and image-based classification in biodiversity monitoring [35, 135]. While these approaches expand analytical capacity [10, 45], their use introduces additional layers of mediation related to model assumptions, training data, algorithmic opacity, and differential performance across languages, ecological contexts, and knowledge systems [19, 37]. Consequently, automation is generally framed as an assistive rather than substitutive component of ethnobiological analysis, requiring sustained interpretative oversight [44, 45].

For the visualization and communication of results, graphical and cartographic tools are commonly employed to translate complex analytical outputs into accessible formats [136, 137]. Interactive maps, dashboards, and network visualizations frequently appear across the studies as interfaces through which analytical results are interpreted, shared, and debated [138, 139]. In participatory contexts, these visualizations are used to support collective interpretation and reflection, enabling engagement by both researchers and community members [14, 140, 141]. Several studies further document the use of visual outputs in environmental education and policy-oriented dialogue, positioning visualization as a mediating practice between scientific analysis, community knowledge, and decision-making arenas [47, 110, 142].

Across the literature, visualization is therefore framed not merely as a communicative output, but as a mediating interface through which analytical results are interpreted, negotiated, and situated within broader socioecological contexts [28, 83]. Comparative analysis indicates that data analysis and visualization tools consistently function as sites where integration and abstraction intersect [43, 136, 137]. Mixed-method analytical workflows are shown to reveal relationships between ecological indicators and cultural values [109, 111], while visual representations simultaneously shape how these relationships are framed and interpreted [143]. Studies caution that analytical outputs and visualizations may privilege certain forms of knowledge, simplify complex local realities, or obscure internal differences and power relations within communities when design choices and interpretative processes are not made explicit [17, 64]. Collectively, these findings indicate that analytical rigor in ethnobiological research using digital technologies depends not only on computational capacity, but also on participatory validation and reflexive engagement with how data are transformed into analytical and visual forms.

Visual outputs are not neutral representations, as design choices related to scale, categorization, aggregation, and graphical emphasis shape how data are interpreted and may reproduce epistemic hierarchies between scientific and traditional knowledge systems if not critically examined [38, 138, 143]. For this reason, participatory validation—through collective interpretation sessions, feedback loops, or iterative redesign—is frequently identified as a key practice for aligning analytical outputs with local perspectives and lived experiences [126, 144].

At the same time, accessibility constraints further condition the use of analytical and visualization tools, with studies documenting barriers related to limited computational infrastructure, unstable internet connectivity, restricted access to software environments, and uneven analytical skills within and across research teams and communities [3, 11, 47, 69]. In response, researchers highlight pragmatic adaptations such as lightweight analytical workflows, offline-capable visualization tools, shared analytical roles within projects, and the prioritization of interpretable models over technically complex but opaque approaches [10, 37, 45]. Rather than eliminating structural inequalities, these strategies are presented as context-dependent adjustments that partially reduce barriers to participation in data-intensive research.

Ethical considerations extend to data transparency, accessibility, and governance, as open-source analytical tools, while promoting reproducibility and collaboration, may also increase the exposure of sensitive ethnobiological information when data governance arrangements are weak or ambiguous [40, 105, 145]. Consequently, clear data-sharing agreements, culturally appropriate metadata practices, and negotiated access controls are emphasized as necessary conditions for protecting community interests while enabling scientific exchange [41, 42].

When embedded within reflexive and participatory research frameworks, digital data analysis and visualization tools support the integration of diverse data types into coherent analytical processes and enable movement beyond descriptive accounts toward interpretative and co-produced knowledge [111, 126]. Their analytical value depends less on technical sophistication than on how analytical authority, representation, and governance are negotiated across scientific, institutional, and community contexts, aligning ethnobiological practice with collaborative and sustainability-oriented research agendas [47, 64].

## Comparative analytical synthesis across digital technology categories

While the previous sections examined digital technologies according to their functional roles within the research process, the comparative synthesis presented here shifts the analytical focus toward cross-cutting dimensions that emerge across these categories. A cross-case comparison indicates that digital technologies used in ethnobiology and ethnoecology exhibit systematic differences in how they shape participation, ethical risk, and epistemological mediation, suggesting that different categories of digital technologies are associated with distinct configurations of visibility, control, and authority in the production, interpretation, and circulation of biocultural knowledge [19, 28, 37, 43, 83].

Three recurrent patterns emerge concerning participation, ethical risk, and methodological trade-offs. First, digital technologies differ in their proximity to local knowledge holders and in their capacity to support participatory engagement: field-based data collection platforms and participatory mapping initiatives tend to involve higher levels of community participation, particularly when data entry, validation, and interpretation are conducted collaboratively [14, 16, 48], whereas remote sensing and large-scale analytical workflows often remain centralized within research teams, with community participation occurring mainly during interpretation or dissemination [112, 119]. Second, digital technologies differ in the types of ethical risks they introduce: participatory mapping and biodiversity cataloging frequently raise concerns related to territorial exposure, intellectual property, and the circulation of culturally sensitive knowledge [15, 41], while remote sensing and automated analytical systems tend to raise concerns related to abstraction, surveillance, and consent at a distance [130, 131]. Third, digital technologies also differ in the balance they establish between standardization and contextual depth: while digital platforms facilitate standardized datasets, interoperability, and cross-site comparison, they may also reduce narrative richness and relational context when not complemented by ethnographic or participatory approaches [10, 44, 64].

These comparative patterns highlight the role of digital technologies as mediating infrastructures that shape participation, ethical risk, and epistemological representation in ethnobiological research. Table 3 summarizes these comparative dimensions and illustrates how different categories of digital technologies tend to cluster around distinct configurations of participation, ethical risk, and analytical mediation.

**Table 3:** Analytical comparison of digital technology categories and their implications for participation, ethics, and knowledge mediation

Technology category	Typical scale	Participation level	Main ethical concerns	Analytical contribution	Epistemological implications
Data collection platforms	Local	Medium – high	Data privacy, consent, ownership of field records	Structured data generation	Standardization of heterogeneous knowledge
Participatory mapping	Local – landscape	High	Territorial exposure, control of spatial data	Spatial documentation of local knowledge	Translation of place-based knowledge into cartographic representations
Biodiversity cataloging	Local – global	Medium	Intellectual property, data sovereignty	Digital archiving of species knowledge	Integration of vernacular and scientific taxonomies
Audio recording / transcription	Local	High	Consent, narrative ownership	Documentation of oral knowledge	Transformation of oral narratives into digital archives
Remote sensing and spatial analysis	Landscape – regional	Low – medium	Surveillance, abstraction of landscapes	Monitoring environmental change	Representation of landscapes as spatial datasets
Data analysis and visualization	Cross-scale	Medium	Representation bias, interpretative authority	Integration and interpretation of datasets	Analytical mediation of socioecological relationships

## Opportunities and benefits

Beyond the comparative patterns identified above, digital technologies also create new opportunities for ethnobiological and ethnoecological research [1, 2, 4]. Empirical and methodological studies document how digitalization has been used to rearticulate scale, participation, and reflexivity within socio-ecological research by enabling connections between localized ethnographic observations and broader spatial or analytical frameworks [31, 69, 110]. Rather than replacing established ethnographic practices, digital approaches expand analytical and communicative capacities when embedded within culturally grounded and participatory methodologies [5, 25].

One recurrent opportunity concerns the capacity of digital infrastructures to connect heterogeneous forms of knowledge, as studies drawing on discussions of information infrastructures and data-centric research show that integrating ethnographic narratives, ecological indicators, and spatial information enables more holistic analyses of biocultural systems [27, 111]. Mixed-method approaches combining qualitative narratives with geospatial or ecological data allow researchers to examine relationships between cultural practices and environmental dynamics that are difficult to capture through single-method approaches, while also facilitating dialogue across disciplinary and epistemic boundaries [10, 107, 109].

A second opportunity relates to participatory engagement across multiple stages of the research process. Case-based studies show that when communities participate not only in data collection but also in interpretation and visualization, digital tools can support dialogical forms of knowledge co-production [14, 126]. Maps, visualizations, and community-accessible databases are frequently used to support collective reflection, local planning, and communication among communities, researchers, and decision-makers [13, 18, 54, 128]. At the same time, studies emphasize that these participatory benefits depend on governance arrangements and cannot be assumed as automatic outcomes of digital tool adoption [47, 64].

Importantly, these opportunities are also accompanied by material, institutional, and political costs, with digital research practices often requiring additional time, technical training, financial resources, and institutional coordination [3, 127]. Empirical accounts highlight trade-offs related to unequal access to digital infrastructure, competing institutional agendas, and internal power asymmetries within communities [69, 129]. In several cases, the pursuit of participatory or open digital practices introduced additional negotiation burdens, delayed research timelines, or generated conflicts over data control and representation [40, 42].

Methodological benefits related to transparency and reproducibility also emerge, as standardized data structures, open analytical workflows, and shared metadata practices enable comparison across sites and research contexts, supporting cumulative knowledge production [10, 45, 46, 127]. Importantly, these benefits do not depend on methodological homogenization but on flexible frameworks capable of accommodating local diversity while maintaining analytical rigor [5, 111].

Overall, the value of digital technologies in ethnobiology and ethnoecology lies less in technological novelty than in their capacity to mediate connections between knowledge systems, analytical scales, and research actors, with these benefits emerging most clearly when digital tools are embedded within ethically grounded, participatory, and reflexive research designs.

## Challenges and limitations

While digital technologies create important opportunities for ethnobiological and ethnoecological research, they also introduce persistent challenges, particularly ethical concerns related to privacy, informed consent, and data sovereignty, which are increasingly recognized as central analytical issues rather than ancillary considerations [23, 24, 67]. Once recorded, digital data can be easily reproduced, circulated, or repurposed beyond their original research context, increasing the risk of misuse when governance arrangements are absent, ambiguous, or unevenly enforced [3, 19, 30].

A second implication concerns the design and governance of digital research processes, as studies emphasize the importance of co-design approaches, particularly in socioecological contexts characterized by cultural heterogeneity, internal power asymmetries, and uneven access to digital resources [22, 126]. Within this perspective, aligning digital research practices with FAIR principles requires careful mediation through CARE-oriented governance frameworks and mechanisms such as biocultural or Traditional Knowledge (TK) Labels, which seek to ensure that interoperability and data reuse remain compatible with community-defined authority, consent, and benefit-sharing arrangements [41, 106, 146]. Empirical studies further emphasize that digital methods tend to be more effective and ethically robust when aligned with locally defined values, capacities, and institutional arrangements rather than being introduced as standardized or externally driven solutions [22, 108].

A third recurrent limitation concerns the potential distortion or decontextualization of cultural knowledge through digital formats, as digital infrastructures may fragment narratives, prioritize standardized categories, or privilege visual and spatial representations over relational and processual meanings embedded in local knowledge systems [11, 37, 143]. These patterns resonate with broader sociotechnical discussions of classification and information infrastructures, which highlight how digital systems shape what can be recorded, stored, and rendered visible [27, 43]. Without careful interpretation and the participatory validation of digital outputs, digital representations may oversimplify complex socioecological relationships or reproduce external analytical frameworks at the expense of local epistemologies [17, 64, 91, 101].

Digital exclusion emerges as a further limitation across the studies, though not as a uniform process, as empirical analyses show that unequal access to devices, connectivity, and technical skills may reproduce existing social inequalities and constrain meaningful participation in digital research processes [11, 147, 148]. Several studies highlight internal differentiation within Indigenous peoples and local communities, indicating that elders, women, or other social groups may be differently affected by digitalization when knowledge transmission practices are not easily translated into digital formats [54, 63, 101, 122, 129]. These findings suggest that digital tools may reconfigure local power relations rather than simply expanding participation.

Challenges related to long-term data maintenance and technological dependence emerge as an additional limitation, as digital platforms require sustained institutional support, continuous updating, and long-term storage infrastructures, raising concerns about the durability of digital archives beyond the lifespan of individual research projects [3, 27, 39]. Addressing these limitations depends not only on technical solutions but also on negotiated agreements, realistic assessments of local capacities, and governance arrangements that define responsibilities for data stewardship over time [22, 42].

Collectively, these challenges indicate that digital technologies do not merely introduce technical constraints but actively mediate ethical responsibilities, epistemological boundaries, and power relations within ethnobiological and ethnoecological research, reinforcing the need for reflexive, context-specific, and participatory approaches to digital research design.

## Implications for methodology and practice

The analyzed studies highlight the relevance of hybrid methodological approaches that combine digital tools with participatory and ethnographic practices, in which digital technologies function not as replacements for established methods but as complementary instruments that support data collection, organization, and analysis while maintaining the contextual depth central to ethnobiological research [5, 25, 126]. Hybrid methodological designs that integrate qualitative narratives, spatial data, and ecological observations are increasingly recognized as particularly suitable for addressing the complexity of socioecological systems and biocultural knowledge dynamics [71, 111].

Another implication concerns the need to design digital research processes through collaborative governance arrangements that involve communities in decisions about data collection, storage, and circulation. Rather than treating digital infrastructures as neutral tools, the reviewed studies indicate that their use requires explicit negotiation of authority, consent, and data stewardship, particularly in socioecological contexts characterized by cultural heterogeneity and uneven access to digital resources [22, 126]. Empirical evidence further shows that digital methods tend to be more effective and ethically robust when embedded within locally defined institutional arrangements and capacities rather than implemented as standardized or externally driven solutions [63, 108, 126].

From a practical perspective, methodological choices in digital ethnobiological research are most productively guided by research objectives and social context rather than by technological availability alone, as studies show that tools perceived as accessible, transparent, and adaptable are more likely to support meaningful participation and mitigate risks associated with exclusion, misrepresentation, or data misuse [16, 48, 51, 57]. At the same time, practices such as layered consent, negotiated data access, community-defined restrictions, and participatory validation of digital outputs are frequently highlighted as mechanisms through which ethical responsibilities and epistemic authority can be actively negotiated in digital research settings [23, 24, 40].

These considerations underscore the importance of reflexive methodological decision-making in digital ethnobiology and ethnoecology, as digital technologies do not merely support research practices but also actively mediate relationships between researchers, communities, and knowledge infrastructures [19, 27]. Embedding digital tools within ethically grounded, context-sensitive, and community-centered research frameworks therefore remains essential for harnessing technological innovation while preserving the epistemological commitments and social responsibilities that define ethnobiological and ethnoecological research.

## Conclusion

Digital technologies are increasingly embedded in ethnobiological and ethnoecological research, shaping how biocultural knowledge is documented, interpreted, and circulated. Rather than functioning as neutral instruments, the literature shows that digital tools operate as mediating infrastructures that influence participation, ethical risk, and epistemological representation across different stages of the research process. Different technological categories are associated with distinct configurations of participation, ethical risk, and knowledge mediation.

These dynamics create important opportunities for integrating heterogeneous knowledge systems, enabling connections between ethnographic narratives, ecological observations, and spatial data. Across the reviewed studies, the comparative analysis conducted in this review also revealed recurring methodological patterns in how digital technologies mediate data collection, participation, and knowledge representation, while simultaneously exposing conceptual tensions related to data governance, cultural appropriateness, and epistemological asymmetries. At the same time, the literature emphasizes that these opportunities are accompanied by persistent challenges related to data governance, digital exclusion, long-term infrastructure maintenance, and the potential decontextualization of culturally embedded knowledge. These findings reinforce that the methodological value of digital technologies depends less on technological sophistication than on the governance arrangements, participatory practices, and interpretative frameworks within which they are embedded.

Future research should therefore move beyond instrumental approaches to digital tools and further explore how digital infrastructures reshape the social and epistemological conditions of ethnobiological research. Advancing this agenda will require interdisciplinary collaboration and sustained engagement with Indigenous and local communities to ensure that digital innovation remains aligned with the ethical commitments and relational principles that define ethnobiology and ethnoecology.

## Policy and practice recommendations

- Co-design digital research tools with Indigenous peoples and local communities
- Apply layered and context-sensitive consent in the collection and sharing of digital data
- Ensure community-defined governance over access to culturally sensitive information
- Combine digital methods with in-person ethnographic engagement to preserve contextual depth
- Select digital tools based on research objectives, accessibility, and ethical risk assessment

## Supplementary Information

Additional file 1.

## Data Availability

Not applicable. All data analyzed in this review derive from publicly available peer-reviewed publications cited in the reference list.
