# Supplementary material for: Digital technologies in ethnobiology and ethnoecology: methodological opportunities, ethical challenges, and participatory implications
Source: J Ethnobiol Ethnomed. 2026 Apr 26;22:65. doi: 10.1186/s13002-026-00889-2 (PMC13267722; doi:10.1186/s13002-026-00889-2)
Supplement: Supplementary file 1 — Additional file 1. [file 13002_2026_889_MOESM1_ESM.docx]

**Supplementary Material S1.** Digital tools used in ethnobiology and ethnoecology, organized by functional category. Functional categories follow the classification framework presented in Table 1 of the main text.

| **Technology** | **Functional category** | **Availability** | **Platform** | **Primary function** | **Applications in ethnobiology and ethnoecology** |
| --- | --- | --- | --- | --- | --- |
| KoBoToolbox | Data collection tools | Free | Web / Android | Structured digital questionnaires | Collection of ethnobiological interview data, species-use surveys, and socio-ecological questionnaires |
| ODK Collect | Data collection tools | Free | Android | Mobile data collection with GPS | Field-based collection of ethnographic, ecological, and spatial data under offline conditions |
| Epicollect5 | Data collection tools | Free | Android / iOS | Form-based data collection with geolocation | Collaborative documentation of species records and local ecological knowledge |
| QField | Participatory mapping | Free | Android | Field-based GIS data collection | Participatory mapping of resource use areas, ecological interactions, and cultural landscapes |
| Maptionnaire | Participatory mapping | Paid | Web / Mobile | Interactive participatory mapping | Collection of spatial perceptions, experiences, and community-based spatial data |
| GPS Essentials | Participatory mapping | Free | Android | GPS tracking and waypoint recording | Documentation of sites of ecological interaction and culturally significant locations |
| iNaturalist | Biodiversity cataloging and documentation | Free | Web / Android / iOS | Collaborative species identification | Documentation of culturally significant species, local taxonomies, and ecological observations |
| Audio Memos | Biodiversity cataloging and documentation | Free | Android | Audio recording and tagging | Recording of interviews, oral histories, and ethnographic narratives |
| Transkriptor | Biodiversity cataloging and documentation | Free (limited) | Web | Automated audio transcription | Transcription and organization of oral data linked to species or ecological contexts |
| Drones | Remote sensing and spatial analysis | Paid | Hardware | High-resolution aerial imagery | Mapping of ecosystems, territories, and land-use patterns relevant to ethnobiological research |
| Satellite imagery (e.g., Sentinel, Landsat) | Remote sensing and spatial analysis | Free / Paid | Web / GIS software | Landscape-scale environmental monitoring | Analysis of vegetation dynamics and environmental change affecting biocultural systems |
| QGIS, R, Python | Data analysis and visualization | Free | Desktop | Spatial, statistical, and network analysis | Integration, analysis, and visualization of socio-ecological and ethnobiological datasets |
| Online biodiversity databases (e.g., GBIF) | Biodiversity cataloging and documentation | Free | Web | Access to biodiversity records | Validation and contextualization of local species records within broader biodiversity datasets |
